# Rapid Reassembly, Biomass-Derived Adhesive Based on Soybean Oil and Diels–Alder Bonds

**DOI:** 10.3390/polym15224428

**Published:** 2023-11-16

**Authors:** Zhiyong Liu, Zhiguo Song, Benrong Lv, Zumin Qiu

**Affiliations:** 1 School of Chemistry and Chemical Engineering, Nanchang University, Nanchang 330031, China; 2 Huzhou Guoneng New Material Co., Ltd., Huzhou 313000, China; 3 Department of Polymer Materials and Engineering, School of Chemical and Environmental Engineering, Anhui Polytechnic University, Wuhu 241000, China

**Keywords:** reassembly adhesive, soybean oil, Diels–Alder, dynamic covalent bonds

## Abstract

Synthetic adhesives play a crucial role in holding together solid materials through interfacial interactions. Thermoplastic and thermosetting adhesives are important types of synthetic adhesives, with thermoplastic adhesives being reassemblable and thermosetting adhesives exhibiting high adhesive strength and creep resistance. However, there is a need to combine the advantages of both types and develop high bonding strength, reassemblable adhesives. Here, epoxidized soybean oil (ESO) was used to prepare adhesive networks and Diels–Alder bonds were incorporated to enhance reassembly ability. The ESO was functionalized with furyl groups and cross-linked via the reaction between furyl and imide groups to involve the Diels–Alder bonds. The resulting adhesive exhibited good solvent resistance and mechanical properties, which could be regulated by adjusting the quantity of cross-linker. The prepared adhesives also demonstrated self-healing capabilities, as the scratch on the surface gradually diminished with heating. Additionally, the adhesives showed the ability to undergo recycling without significant changes in properties. The prepared adhesives exhibited hydrophilicity and the flow characteristics during reassembly were characterized by a decrease in torque. This study provides a promising approach for the development of synthetic adhesives with reassembly ability, which has important implications for the field of bonding.

## 1. Introduction

Adhesives can rely on interfacial interactions to hold together the same or different kinds of solid materials. Adhesives play an irreplaceable role in improving the quality of human life, and adhesive technology, along with its unparalleled special processes, plays a significant role in modern economy, modern technology, and even modern defense. Adhesives can be classified into two main categories: natural adhesives and synthetic adhesives. Natural adhesives can be further divided into plant-based adhesives, animal-based adhesives, and mineral-based adhesives. There are various types of plant-based adhesives, including starch-based adhesives, lignin-based adhesives, soy protein-based adhesives, dextrin-based adhesives, cellulose-based adhesives, and so on. Most biomass-based adhesives can be classified as plant-based adhesives, hence research on plant-based adhesives is quite common. Natural adhesives have been used for thousands of years and are the oldest type of adhesive. This is mainly due to the easy availability of raw materials, simple manufacturing processes, and convenient use. In an era where industrial technology was less advanced, natural adhesives naturally became indispensable materials in production and daily life. However, the raw materials for natural adhesives are limited by natural conditions, and their bonding strength is low, making them unsuitable for modern production. Additionally, natural adhesives, especially biomass-based adhesives, generally have poor water resistance, leading to their gradual replacement by synthetic adhesives. The molecular structure of synthetic adhesives is highly designable, and adhesives with different properties can be prepared by adjusting the types and proportions of components to meet the needs of different fields [1,2,3,4,5,6,7]. Therefore, the synthetic adhesives have been widely used and the demand is increasing. Thermoplastic and thermosetting adhesives play an important role in synthetic adhesives. Thermoplastic adhesives are made of thermoplastic resin such as polymethacrylate, polyvinyl, etc. In a certain temperature range, their physical state changes with temperature while the chemical characteristics remain unchanged. The curing process does not include any chemical reaction, only through the solvent volatilization or melting–cooling. Therefore, thermoplastic adhesives could be reassembled according to the intention which provides a wider operating time window and a higher fault tolerance rate for practical applications. However, their creep resistance and mechanical strength are limited.

Compared with thermoplastic, thermosetting adhesives (such as phenolic resin and epoxy resin) exhibit high adhesive strength and creep resistance owing to the intrinsic cross-linked networks. However, the covalent cross-linked networks inside thermosetting adhesives typically require external stimuli to be generated, and, due to the time required for the cross-linking curing reaction, it brings many inconveniences to the application process, especially when the adherend materials are unstable. In addition, the cross-linking curing reaction forms an insoluble and non-melting adhesive layer, which cannot be reused, resulting in a lower fault tolerance rate and higher precision requirements during application. Therefore, when combining the advantages of the two kinds of synthetic adhesives and prepare high bonding strength, reassembled adhesives are of great significance to the development of the field of bonding.

In order to achieve this objective, researchers have made many meaningful attempts. Currently, there are several types of adhesive with reassemblable properties, including thermoplastic adhesives, degradable adhesives, photoinduced reversible adhesives, and thermally induced reversible adhesives. As mentioned earlier, thermoplastic adhesives lack cross-linking structures, resulting in poor thermal stability and dimensional stability. Although photocontrolled adhesives can achieve disassembly and reassembly, their adhesive strength is relatively weak. Degradable adhesives often only allow for one-time bonding and disassembly, lacking reusability. Among these, introducing dynamic covalent bonds into adhesives cross-linked networks is an approach with great potential [8,9,10,11]. Dynamic covalent bonds generally refer to a class of chemical bonds or reversible reactions which can undergo exchange reactions under external stimulation, including Diels–Alder bonds [12,13,14], imines [15,16], disulfides [17,18,19], transesterification [20,21], transamination [22,23], hindered urea bonds [24,25,26], dynamic amides [27], boroxines [28,29], and so forth. Due to their stability and dynamic nature, the incorporation of dynamic covalent bonds into polymer materials can impart self-healing, reprocessability, and recyclability properties to the materials. The introduction of dynamic covalent bonds into adhesive systems can confer removability and reusability to the adhesives, which is of great practical significance for the development of environmentally friendly and recyclable adhesives. Although there are various dynamic covalent bonds, taking advantage of appropriate dynamic covalent bonds is particularly important.

On the basis of exchange mechanism, dynamic covalent bonds could be divided into associative and dissociative bonds [30,31]. The exchange reaction of associative bonds likely occurs at room temperature with a very slow rate. The external stimulation could accelerate the reaction rate, but the activation energy of the reaction is not affected. The networks containing associative bonds have a constant cross-linking density during the rearrangement process of the network topology. Therefore, the viscous fluidity is determined by the exchange rate and makes it difficult to achieve a low viscosity as thermoplastics. This is unfavorable to the requirement of fast reassembly of adhesives in a short time in practical application. In contrast, the dissociative bonds are first broken and then reformed in the rearrangement process of the network topology, so the network exhibits low viscosity. Diels–Alder bonds are a typical representative of the dissociative bonds, and its dissociation mechanism has been well studied.

Moreover, the raw materials of synthetic adhesives are mostly petroleum-based. With the depletion of petroleum resources and the aggravation of environmental problems, the preparation of synthetic adhesives from biomass resources has attracted great attention [32,33,34,35]. In terms of price and production scale, palm oil, castor oil, and soybean oil are the most promising vegetable oils. Palm oil is mainly produced in Southeast Asia, and has the largest yield. However, its production has a certain seasonality and palm oil-based polyols are not resistant to low temperature which is inconvenient for transportation and storage, limiting its application. Because the global production of castor oil less than 1.5 million tons, the cost advantage is not obvious. The global production of soybean oil is over 60 million tons. The harvest is not seasonal and the supply is stable throughout the year. Therefore, soybean oil will remain the most important raw material in the future development of vegetable oil industry. There are many advantages of using epoxy soybean oil for the preparation of synthetic adhesives. Firstly, it is a green and environmentally friendly alternative as it is derived from renewable plant resources. Secondly, epoxy soybean oil possesses excellent bonding properties and can firmly bond various different materials, such as metals, plastics, and wood. Additionally, it has good heat resistance and chemical resistance, allowing it to maintain stable adhesive effects in high temperature and corrosive environments. Moreover, epoxy soybean oil adhesives have a longer open time and faster curing speed, improving production efficiency. In summary, epoxy soybean oil as an adhesive has the advantages of being environmentally friendly, having strong bonding properties, good heat and chemical resistance, and high efficiency.

Here, epoxidized soybean oil (ESO) was used as the synthetic adhesives’ substrate, and Diels–Alder bonds were introduced to endow the prepared adhesives with reassembly ability. Owing to the fact that Diels–Alder bonds are dissociatived dynamic covalent bonds, the viscosity of the cross-linked network topology decreased rapidly during rearrangement, endowing the adhesive with the ability to quickly reassemble.

## 2. Experimental Section

### 2.1. Materials

Epoxidized soybean oil (ESO) was lab-made and characterized [36]. Furfuryl mercaptan, lithium hydroxide, and bismaleimide (BMI) were supplied by Adamas-beta (Shanghai, China). All other reagents were commercial chemicals and used as received without further purification.

### 2.2. The Modification of ESO with Furyl Groups (ESOF)

Here, 10 g epoxidized soybean oil, 10 g furfuryl mercaptan, and 0.8 g lithium hydroxide were mixed in a flask equipped with a magnetic stir. Then, the mixture was heated at 100 °C for 6 h under nitrogen atmosphere. As the reaction progressed, the viscosity of the mixture increased, and the color turned dark yellow. At the end of the reaction time, the mixture was precipitated in a large amount of petroleum ether and washed several times. The precipitate was dried in a vacuum oven to obtain the target product which denoted as ESOF for convenience.

### 2.3. The Preparation of Cross-Linked ESO-Based Films

The cross-linked ESO-based films were prepared through a casting into strategy. In brief, 3 g ESOF was first dissolved in 20 mL dimethylacetamide, followed by 1.5 g bismaleimide was added. Then the solution was cast into a PTFE mold (10 × 10 × 1 cm^3^) and placed in the oven for 4 d at 80 °C to obtain a cross-linked film. For convenience, the film was denoted as ESOF-I_1.0_ in which the number 1.0 represented for the mole ratio of imide groups and furyl groups in the preparation. Similarly, the other two films ESOF-I_0.8_ and ESOF-I_0.6_ could be prepared according to a similar preparation process.

### 2.4. Recycling of Cross-Linked ESO-Based Films

The recycling experiments were conducted on a vulcanizer (Hartek, Guangzhou, China). Due to the ESOF-I_1.0_ film having the highest cross-linking density, it was chosen as the typical material for this experiment. Firstly, the ESOF-I_1.0_ film was sheared into irregular fragments with a length and width of approximately 1 cm, and then the fragments were collected and put into a steel mold on a press vulcanizer under 10 MPa at 130 °C for 1 h to form new samples. The sample dimension was 5 × 5 × 1 cm^3^. The process was repeated for three times and each recycled sample were tested using electronic universal testing machine and differential scanning calorimetry.

### 2.5. Characterizations

Differential scanning calorimetry (DSC) analyses were performed on DSC 25 (TA Instruments, New Castle, DE, USA) under nitrogen atmosphere at a heating or cooling rate 20 °C/min. The samples were heated from room temperature to 80 °C and then cooled down to −80 °C to eliminate the thermal history.

The ultraviolet-visible spectroscopy tests were conducted on UV-2550 spectrophotometer (Shimadzu, Kyoto, Japan), the dichloromethane was used to dissolve the substance to be measured.

Proton nuclear magnetic resonance spectroscopy (^1^H NMR) was performed using a Brucker 500 MHz Avance (Bruker BioSpin Corp., Billerica, MA, USA) to elucidate the structural characteristics. Unless otherwise specified, the spectra were obtained at room temperature using deuterated chloroform as the solvent. Chemical shifts were referenced to tetramethylsilane (TMS).

The swelling experiments were conducted as follows: a piece of film (m_0_ g) was immersed in dimethylacetamide (20 mL) for 3 d, then the swelling film was wiped softly by filter paper and weighted (m_1_ g). Lastly, the swelling film was dried in an oven at 80 °C to a constant weight (m_2_ g). The swelling ratio (SR) and the gel content (GF) were calculated according to the following equations:

$$SR=\frac{m_{1}-m_{2}}{m_{2}}$$

$$GF=\frac{m_{2}}{m_{0}}$$

The mechanical properties of prepared films were tested with an electronic universal testing machine (Instron Corporation, Norwood, MA, USA) at a crosshead speed of 50 mm min^−1^. Dumbbell-shaped specimens were cut according to GB/T528 (overall length: 50 mm; inner width: 4 mm). At least five specimens per experimental point were tested in all mechanical measurements to obtain reliable values.

The water contact angel was measured on optical contact angel meter and interface tenslometer (SL250, KINO Industry Limited, Somerville, MA, USA) and fitted by Young–Laplace equation. A brief description of the instructions are as follows: prepare the solid surface by thoroughly cleaning and drying it to remove any contaminants or impurities that may affect the wetting behavior. Place the cleaned solid surface on a flat and level platform, ensuring stability and accuracy during the experiment. Dispense a small droplet of water onto the solid surface. Care should be taken to ensure that the droplet size is consistent and reproducible. Allow the droplet to settle on the solid surface until it reaches equilibrium, ensuring that there is no visible motion or deformation of the droplet. This ensures that the contact angle measurement is accurate. Using a high-resolution camera or a microscope, capture an image of the droplet on the solid surface from a suitable angle. This image will later be analyzed to determine the contact angle. Repeat the experiment at least three times for each solid surface to ensure reproducibility and obtain an average contact angle value.

The torque of prepared adhesive at elevated temperature was recorded using RPA 8000 A (Gotech Testing Machines Inc., Dongguan, China).

## 3. Results and Discussion

**Scheme 1:**
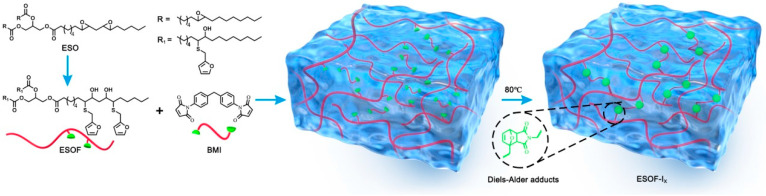
The synthesis route of ESOF-I_x_. x is the mole ratio of imide groups and furyl groups.

**Figure 1:**
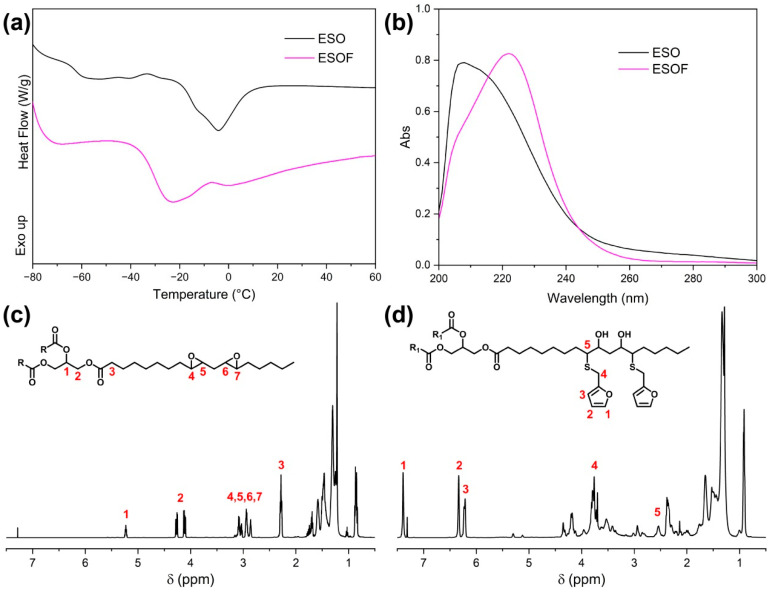
(**a**) The comparison DSC curves, (**b**) ultraviolet-visible spectra of ESO and ESOF. The ^1^H NMR spectra of (**c**) ESO and (**d**) ESOF.

The synthesis route of ESOF was depicted in the Scheme 1. On the basis of the reaction between the thiol and epoxy group, the furyl group could be grafted onto the ESO chains. Therefore, the thermal performance of ESO and ESOF would be different. Their DSC curves were presented in Figure 1a. In the ESO curve, a distinct endothermic peak was observed at approximately −3.3 °C, indicating the melting points of ESO at this temperature. In contrast, ESOF exhibited a melting point at around −23.7 °C, suggesting that the introduction of the furyl group increased the distance among chains and the weakened intermolecular interactions reduced the melting temperature. This shift in melting temperature could be an evidence of modified ESO. Compared to ESO, the unsaturated double bonds in the furyl group of ESOF can undergo π→π* transitions, resulting in a redshift of the ultraviolet absorption peak. This can be confirmed through ultraviolet-visible spectroscopy tests as shown in Figure 1b. The maximum absorption wavelength changed from 207 nm to 223 nm indicating the incorporation of the furyl group into the main chain of ESO. The chemical structures of ESO and ESOF were analyzed by ^1^H NMR. In Figure 1c, the peaks around 3.0 ppm indicated the existence of epoxy group. Moreover, it could be calculated that there were four epoxy groups of each chain based on the integral of these peaks. In comparison, characterization peaks of furyl group were shown in Figure 1d at 6.2 ppm, 6.3 ppm, and 7.4 ppm. The peaks at 3.0 ppm mostly disappeared indicating epoxy groups had successfully undergone a ring-opening reaction. According to the comprehensive test results, it can be concluded that the ESOF has been successfully prepared according to the designed modification route.

**Figure 2:**
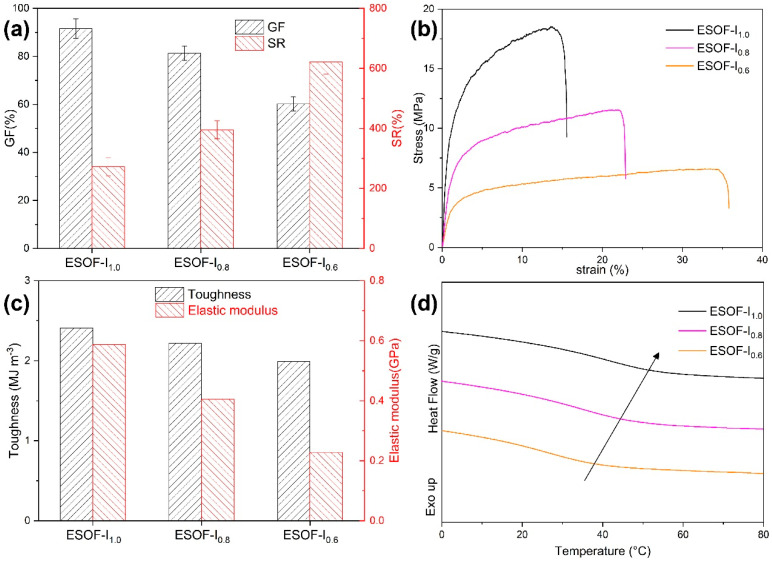
(**a**) The swelling results, (**b**) the stress–strain curves, (**c**) toughness and elastic modulus, (**d**) DSC curves of ESOF-I_x_.

The construction of the covalent polymer network was carried out according to the procedure depicted in Scheme 1. Bismaleimide (BMI) was utilized as the cross-linker and mixed with ESOF initially. Subsequently, the polymer networks, designated as ESOF-I_x_, were gradually formed upon heating at 80 °C due to the formation of Diels–Alder adducts between the furyl and imide groups. To confirm the formation of covalent cross-links, swelling tests were conducted. The resistance of the obtained films to solvents was assessed using dimethylacetamide. The swelling ratio (SR) and gel fraction (GF) were determined and depicted in Figure 2a. The results revealed that the GF and SR of ESOF-I_0.6_ were approximately 60% and 621%, respectively. However, when the amount of BMI used was increased, the GF of ESOF-I_1.0_ increased to 92%. This observation suggests that a higher amount of BMI resulted in an elevated cross-linking density, thereby enhancing the solvent resistance of the prepared adhesives.

The quantity of cross-linker plays a significant role in determining the properties of the adhesive, which is crucial for practical applications. Consequently, the obtained films were subjected to tensile tests, and the resulting curves are illustrated in Figure 2b. Evidently, by adjusting the quantity of BMI utilized, the mechanical properties of the adhesive could be regulated. For instance, ESOF-I_1.0_ exhibited a higher stress at break (18.45 MPa) compared to ESOF-I_0.6_ (6.53 MPa) due to the increased utilization of BMI during the preparation process. Furthermore, the toughness of ESOF-I_1.0_, as determined by the integral area of the curves, was approximately 2.4 MJ m^−3^, whereas ESOF-I_0.6_ only exhibited 1.9 MJ m^−3^ (Figure 2c). In addition, the elastic modulus also decreased from 0.58 GPa to 0.22 GPa, indicating that the adhesive softened as the crosslinking density decreased. The tensile results indicated that the mechanical properties of the adhesive could be customized to meet specific requirements. Additionally, the glass transition temperature (*T*_g_) gradually increased with the quantity of BMI, as observed from the DSC results (Figure 2d). For example, ESOF-I_0.6_ transitioned from a glass state to a rubber state at around 34.6 °C, whereas the *T*_g_ of ESOF-I_1.0_ was approximately 47.3 °C, attributed to the strengthened interaction between molecular chains with the increased amount of BMI.

**Figure 3:**
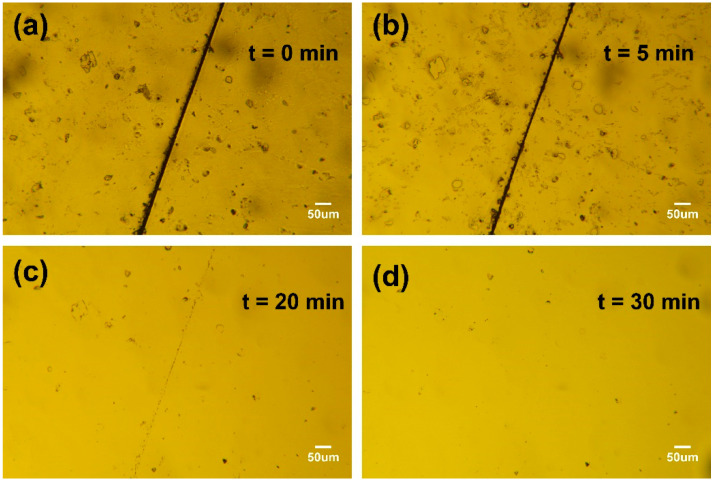
The digital images of cracked ESOF-I_1.0_ under heat (**a**) 0 min, (**b**) 5 min, (**c**) 20 min and (**d**) 30 min.

The decline in adhesive performance is often caused by internal cracks, which are difficult to detect and repair in a timely manner. This leads to a reduction in mechanical performance and a shortened service life of adhesives, restricting their application range. Therefore, it is crucial to repair cracks promptly and achieve self-healing of adhesives, as it significantly improves the utilization efficiency of materials. Self-healing adhesives are capable of perceiving changes in the external environment and responding accordingly, ultimately restoring their own performance. They are a widely applicable and highly demanded type of intelligent material. In the case of ESOF-I_x_, intrinsic self-healing properties are expected due to the incorporation of Diels–Alder bonds. To evaluate the self-healing capability, ESOF-I_1.0_ was intentionally scratched to create a surface crack, and then subjected to heating at 140 °C for different durations. As illustrated in Figure 3, the crack gradually diminished as the heating time increased, demonstrating remarkable self-healing performance. This phenomenon is attributed to the ability of Diels–Alder bonds to be disconnected at high temperatures, thereby disrupting the cross-linked network structure and increasing the mobility of the chains. Consequently, the chains are able to move between the two interfaces of the scratch. Upon cooling, the Diels–Alder bonds can reform; however, due to the altered relative position of the chains, the structure of the cross-linked network is reconstructed, effectively repairing the scratch.

**Figure 4:**
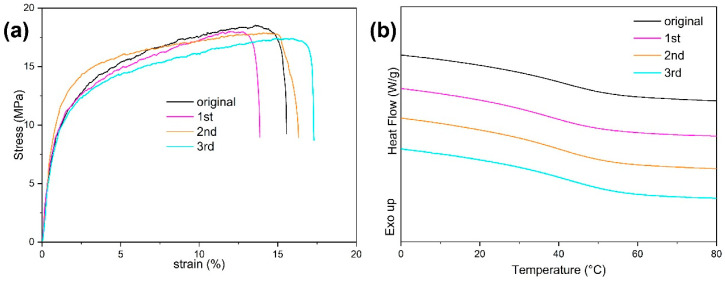
(**a**) The stress–strain curves and (**b**) DSC curves of original and recycled ESOF-I_1.0_.

In addition, the inclusion of dynamic Diels–Alder bonds bestowed ESOF-I_x_ with the ability to undergo recycling. When fragments of ESOF-I_1.0_ film were subjected to hot pressing at 130 °C and 10 MPa for 1 h, an intact film without any visible defects was obtained. This recycling process could be repeated up to three times, and subsequent tensile tests were conducted to evaluate the efficiency of the recycling. As depicted in Figure 4a, the stress–strain curves of ESOF-I_1.0_ displayed a similar pattern after each cycle. After subjecting the adhesive to three cycles of hot pressing, DSC tests revealed that the *T*_g_ did not undergo significant changes in comparison to the original sample, indicating that the cross-linking density remained constant while the network structure was adjusted (Figure 4b). The ability to retain properties in the recycled networks can be attributed to the abundance of dynamic Diels–Alder bonds, which facilitate the reorganization of the network topology.

**Figure 5:**
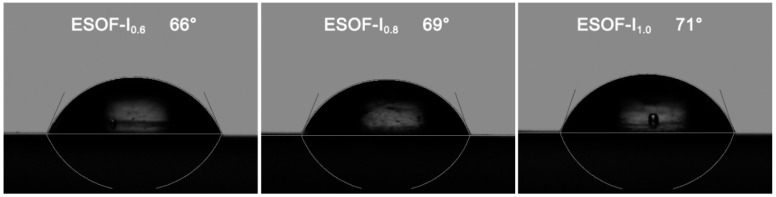
The digital photos depicting water contact angle experiments of the prepared ESOF-I_x_.

Adhesion is the result of the interaction between different materials at their interface. Therefore, the role of the interface layer is a fundamental issue in the study of adhesive science. Factors such as interfacial tension, surface free energy, functional group properties, and interfacial reactions between the adherend and adhesive all influence the adhesive strength. In order to achieve a good bonding effect, the adhesive must have the ability to wet the surface of the adherend, ensuring sufficient contact between the adhesive and the adherend. When the surface of the adherend has uneven topography, the wetting and spreading of the adhesive can fill in the peaks and valleys, allowing the two surfaces to come into full contact, thereby achieving the optimal distance for intermolecular forces to act. The wetting ability can be measured by the contact angle, which is the angle between the tangent line of the liquid surface at the solid–liquid–gas three-phase contact point and the solid plane on the side where the liquid droplet contacts. Generally, a contact angle smaller than 90 degrees indicates a wetting state, and the smaller the contact angle, the better the wetting performance, facilitating the binding of interfaces through supramolecular interactions. Here, the wetting ability of the prepared adhesives was analyzed using water contact angle experiments. As shown in the Figure 5, the water contact angles of the prepared adhesives are all less than 90 degrees, indicating their good wetting ability. This is mainly due to the presence of polar groups such as hydroxyl groups, esters, and imides in the networks. Furthermore, as the cross-linking density increases, the water contact angle slightly increases, which may be attributed to the tighter cross-linked network structure.

**Figure 6:**
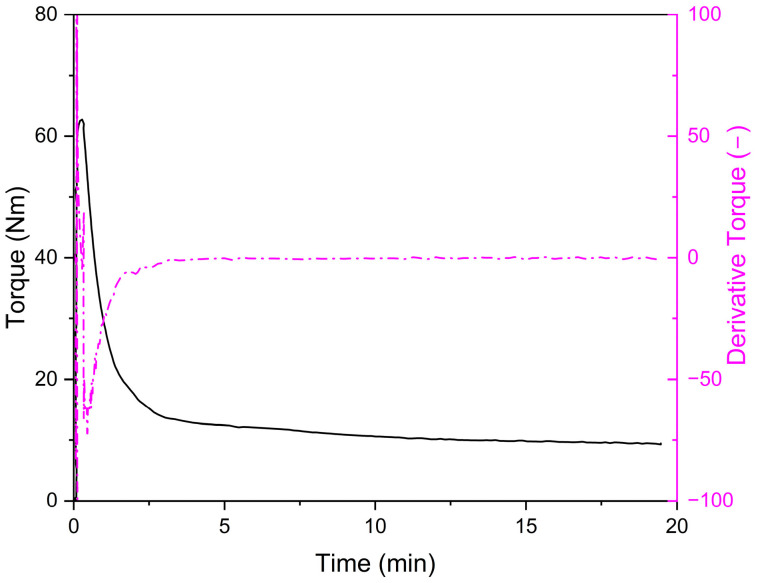
The torque and derivative torque curves of ESOF-I_1.0_ over time at 140 °C.

To evaluate the flow characteristics of adhesives during the reassembly process, a vulcanization analyzer was utilized to monitor the changes in torque of the adhesive over time at a specific temperature. The findings, as shown in Figure 6, indicated that the torque initially increases rapidly, which can be attributed to the precipitate contact between the adhesive and the rotating plate. Subsequently, the torque decreases quickly due to the softening of the adhesive upon heating. Finally, the thermal dissociation of Diels–Alder bonds leads to the gradual collapse of the cross-linked network, resulting in the transformation into linear molecular chains. The torque continued to decrease until it reached a stable state. Since torque is indicative of viscosity, these experimental results demonstrated that the viscosity of the cross-linked network topology can rapidly decrease during rearrangement at elevated temperatures, thus facilitating the reassembly of adhesives. This phenomenon could be attributed to the dissociation of dynamic covalent bonds.

## 4. Conclusions

This study aimed to modify and develop ESO to create dynamic covalent networks incorporating dynamic Diels–Alder bonds. The successful synthesis of ESOF was confirmed through DSC curves, ultraviolet-visible spectra, and ^1^H NMR results. Swelling experiments revealed that the cross-linked networks were constructed and exhibited good resistance to solvents. The mechanical properties of the networks including stress and strain at break were influenced by the molar ratios between BMI and ESOF, ranging from 6.53 MPa to 18.45 MPa. The *T*_g_ values of the networks, determined by DSC, ranged from 34.6 °C to 47.3 °C. Self-healing and recycling experiments demonstrated that the networks underwent topology rearrangement at high temperatures. The water contact angle experiments indicated the potential application of the prepared material as adhesives due to the presence of polar groups. Furthermore, the networks exhibited low viscosity as the dissociation of Diels–Alder bonds at elevated temperatures, facilitating the reassembly of broken interfaces. This renewable adhesive derived from biomass possesses self-healing and reassembly abilities, contributing to reduced energy consumption and environmental pollution.

## Data Availability

The data presented in this study are available upon request from the corresponding authors.
